# Differential Proinflammatory Responses to *Aspergillus fumigatus* by Airway Epithelial Cells In Vitro Are Protease Dependent

**DOI:** 10.3390/jof7060468

**Published:** 2021-06-10

**Authors:** Jessica Rowley, Sara Namvar, Sara Gago, Briony Labram, Paul Bowyer, Malcolm D. Richardson, Sarah E. Herrick

**Affiliations:** 1 School of Biological Sciences, Faculty of Biology Medicine and Health, University of Manchester and Manchester Academic Health Science Centre, Manchester M13 9PT, UK; j.rowley@imperial.ac.uk (J.R.); s.namvar@salford.ac.uk (S.N.); sara.gago-2@manchester.ac.uk (S.G.); briony.labram@nc3rs.org.uk (B.L.); paul.bowyer@manchester.ac.uk (P.B.); Malcolm.Richardson@manchester.ac.uk (M.D.R.); 2 School of Science, Engineering and Environment, University of Salford, Salford M5 4WT, UK; 3 Manchester Fungal Infection Group, Division of Infection, Immunity and Respiratory Medicine, University of Manchester, Manchester M13 9NT, UK; 4 NIHR Manchester Biomedical Research Centre, Manchester Academic Health Science Centre, Manchester University NHS Foundation Trust, Manchester M23 9LT, UK; 5 Mycology Reference Centre, ECMM Excellence Centre of Medical Mycology, Manchester University NHS Foundation Trust, Manchester M23 9LT, UK

**Keywords:** *Aspergillus fumigatus*, airway epithelium, proteases, inflammatory cytokines, fungal lung disease

## Abstract

*Aspergillus fumigatus* is an important human respiratory mould pathogen. In addition to a barrier function, airway epithelium elicits a robust defence against inhaled *A. fumigatus* by initiating an immune response. The manner by which *A. fumigatus* initiates this response and the reasons for the immunological heterogeneity with different isolates are unclear. Both direct fungal cell wall–epithelial cell interaction and secretion of soluble proteases have been proposed as possible mechanisms. Our aim was to determine the contribution of fungal proteases to the induction of epithelial IL-6 and IL-8 in response to different *A. fumigatus* isolates. Airway epithelial cells were exposed to conidia from a low or high protease-producing strain of *A. fumigatus,* and *IL-6* and *IL-8* gene expression and protein production were quantified. The role of proteases in cytokine production was further determined using specific protease inhibitors. The proinflammatory cytokine response correlated with conidia germination and hyphal extension. IL-8 induction was significantly reduced in the presence of matrix metalloprotease or cysteine protease inhibitors. With a high protease-producing strain of *A. fumigatus,* IL-6 release was metalloprotease dependent. Dectin-1 antagonism also inhibited the production of both cytokines. In conclusion, *A. fumigatus*-secreted proteases mediate a proinflammatory response by airway epithelial cells in a strain-dependent manner.

## 1. Introduction

*Aspergillus fumigatus* is the causal agent of aspergillosis, a pulmonary disorder which globally affects over 14 million people [1,2]. It has been estimated that individuals inhale several thousand fungal conidia per day, with higher concentrations linked to the occurrence of aspergillosis [3]. In the healthy host, inhaled *A. fumigatus* conidia are efficiently removed from the airways by the lung defence system. However, in some patients with an impaired immune system, a previous cavitating lung infection or a chronic respiratory condition [4,5], *A. fumigatus* conidia can elude the host immune response, persist and germinate in the lungs, promoting the development of fungal disease.

The epithelial lining of the airway is the first line of defence to inhaled conidia, and as well as acting as a physical barrier, it is paramount in orchestrating a robust innate defence [6]. In vitro, *A. fumigatus* conidia cultured with airway epithelial cells demonstrate limited germination when internalised compared with non-internalised conidia [7,8,9]. Additionally, evidence points to a pivotal role for *A. fumigatus*-exposed airway epithelium in directing inflammatory responses including regulation of the cytokine signature [10,11,12,13], generation of reactive oxygen species [14] and defensins production [15], thereby contributing to the pathogenesis of airway disease [16]. Of note, inactivated and irradiated conidia that are unable to germinate do not elicit cytokine induction, suggesting that germination is a key step in the initiation of an epithelial innate immune response [13,17]. In vitro, proinflammatory mediators, including IL-6 and IL-8, are produced by airway epithelial cells in response to *A. fumigatus* [13,14,17,18,19,20] and are known to mediate mucus secretion [21], sub-epithelial fibrosis [22], neutrophil recruitment and Th2-type immune response [20,23]. IL-6 levels are elevated in asthmatic sputum samples [24], whilst IL-6 deficiency in mice is associated with increased susceptibility to aspergillosis [25]. Furthermore, high IL-8 levels are associated with asthma exacerbations [26] and raised IL-8 levels in lung lavage are an emerging biomarker for invasive aspergillosis [27], which also correlate with neutrophilia and declining lung function in allergic bronchopulmonary aspergillosis [28].

Previous studies have focused on the role of innate immune cells, including macrophages, neutrophils and dendritic cells, in the recognition of and response to germinating *A. fumigatus* conidia [29]. Cytokine secretion by airway macrophages in response to germinating conidia is dependent on the morphological stage of growth with swollen conidia and germlings in particular, inducing a robust inflammatory response predominately through the C-type lectin receptor, Dectin-1 [30]. β-glucan, a structural cell wall glycoprotein present on swollen conidia and hyphae of *A. fumigatus*, is the principal ligand recognised by Dectin-1 on inflammatory cells, and this interaction is essential for fungal phagocytosis, cytokine production and clearance [31,32]. In a similar manner to inflammatory cells, airway epithelial cells also express Dectin-1 and its interaction with β-glucan is proposed to be pivotal to *A. fumigatus* conidia internalisation and cytokine production [12,14,33]. Less is known, however, regarding the contribution of *A. fumigatus* proteases that are secreted during fungal growth and their induction of proinflammatory cytokine production by airway epithelium [34,35,36,37]. Importantly, such fungal-derived proteases have been associated with mediating an allergic response and airway hyper-responsiveness in experimental rodent models [38,39] and their presence in human airways is associated with increasing asthma severity [40]. We previously reported that *A. fumigatus* grown in protein-rich culture media secrete a high level of proteases with dominant serine and metalloprotease activity compared with when grown in minimal Vogel’s media [41]. Furthermore, we demonstrated that the administration of *A. fumigatus* culture filtrate intranasally in mice mediated a Th2 allergic response, and fungal proteases played a major role in the induction of airway wall remodelling [42]. However, the interplay between fungal proteases secreted during conidia germination and hyphal growth and fungal cell wall activation of pattern recognition receptors (PRRs), such as Dectin-1, is likely to be complex and possibly interlinked [43]. For instance, cleavage of neutrophil Dectin-1 receptor by serine proteases produced by *A. fumigatus* results in diminished anti-fungal immunity [44]. Therefore, both direct host–fungal interactions and secreted fungal proteases may be important for the induction of proinflammatory cytokines by airway epithelial cells. In the current study, we analysed the relevance of secreted fungal proteases to the production of IL-6 and IL-8 by airway epithelial cells exposed to germinating conidia from both high and low protease-producing *A. fumigatus* isolates, and we show that epithelial inflammatory responses depend on both secreted protease activity and Dectin-1–β glucan interactions.

## 2. Materials and Methods

### 2.1. A. fumigatus Culture

*A. fumigatus* strain, Af293 (low protease producer in minimal media [41]), was a gift from the Mycology Reference Centre, (Wythenshawe Hospital, Manchester, UK) and strain A1160pyrg+ (high protease producer in minimal media), derived from a parent clinical isolate (CEA10), was a gift from Dr. M. Bromley (University of Manchester, Manchester, UK). Green fluorescent protein-expressing *A. fumigatus* (GFP-AF) was a gift from Professor M. Moore (Simon Fraser University, Burnaby, BC, Canada). *A. fumigatus* strains were propagated on Sabouraud dextrose agar (Oxoid, Basingstoke, UK) at 37 °C for 48–72 h. Conidia were harvested by gentle agitation in sterile phosphate buffered saline (PBS)/0.05% Tween 20, filtered through 4 layers of Whatman filter paper to remove hyphal fragments and counted. The protease activity of the epithelial cell culture supernatant containing Af293 and A1160pyrg+ conidia, compared to *A. fumigatus* culture filtrates following growth in Vogel’s minimal media, was determined as previously described using a universal protease substrate (Casein, resorufin-labelled; Roche, Sussex, UK) according to the manufacturer’s instructions [41,42].

### 2.2. Epithelial Cell Culture, Conidia Germination and Growth

Human bronchial epithelial cells (16HBE14o-) were provided by Dr. Dieter Gruenert, University of California San Francisco [45]. 16HBE14o-cells were seeded into 12-well plates at 1.5 × 10^5^ cells/mL in minimal essential media (MEM; ThermoFisher, Paisley, UK) supplemented with 10% fetal bovine serum (FBS), 2 mM L-glutamine and 1% *v/v* penicillin/streptomycin (PAA, Yeovil, UK). When 80% confluent, cells were washed and cultured overnight in serum-free supplemented MEM. In the initial dose-response experiments, epithelial cells were exposed to a rising concentration of Af293 *A. fumigatus* conidia, ranging from 10 to 10^6^ conidia. Thereafter experiments were conducted using a total of 10^6^ Af293, A1160pyrg+ or GFP-*A. fumigatus* conidia, which were washed and applied to cell monolayers or cell-free wells incubated at 37 °C in 5% CO_2_. For automated, live-cell, time-lapse imaging, an AS MDW live cell imaging system was used with a 20× HC Plan Fluotar objective with a working distance of 1.15 mm, and a green (GFP) LED fluorescent light source in conjunction with the imaging software Image Pro 6.3. Images were captured every 0.5 h for 24 h using a Cascade II EM CCD camera for ultra-sensitive imaging with four images taken per well.

### 2.3. Protease Inhibition and Dectin-1 Receptor Inhibition

16HBE14o-cells were seeded in 24-well plates at 1.5 × 10^5^ cells/mL as described above. Conidia were washed and diluted to the appropriate concentration and applied to cell monolayers for specified times. For conditioned media transfer studies, media was collected from 24 h cultures, filtered through Millex^®^ 0.22 μm syringe-driven filter units (Millipore, Watford, UK) to remove conidia and fungal fragments and applied to fresh serum-starved 16HBE14o- cell cultures for a further 24 h. Protease inhibitors including serine protease inhibitors, antipain (10 μg/mL), matrix metalloprotease (MMP) inhibitor, ilomostat (2.5 μM) and cysteine protease inhibitor E64 (10 μM) were added to serum-starved 16HBE14o- for 15 min prior to *A. fumigatus* exposure. All inhibitors were purchased from Sigma-Aldrich, Poole, UK. To determine the role of Dectin-1, laminarin—a soluble, linear β-glucan from the marine algae *Laminaria digitata* (Sigma-Aldrich)—was applied at a final concentration of 10 mg/mL to serum-starved cultures 30 min prior to the addition of *A. fumigatus*.

### 2.4. Human Nasal Epithelial Cell Culture

Primary human nasal epithelial cells (HNECs) were purchased from PromoCell (Heidelberg, Germany) and cultured at passage two in the proliferation media (PromoCell) until >90% confluent. For air–liquid interface (ALI) studies, HNECs were seeded at a density of 16.5 × 10^4^ cells/mL in 0.5 mL differentiation media (PromoCell) in 12 mm Transwell^®^ plates (7.4 × 10^4^ cells/cm^2^) with 0.4 µM pore polyester membrane inserts and differentiation media in the basal compartment. When confluent, apical media was removed to facilitate ALI (day 0), where cells differentiate and become ciliated and mucus secreting. Cells were maintained at ALI until day 14, at which point they were exposed to *A. fumigatus* (Af293) conidia in a similar manner to submerged 16HBE14o- cells.

### 2.5. Cytokine Gene Expression and Protein Production

For mRNA analysis, cell layers were collected in 300 μL RNAprotect Cell Reagent (Qiagen, Crawley, UK) and frozen at −80 °C. Qiagen’s RNeasy Plus Mini Kit was used to extract RNA according to the manufacturer’s instructions. Applied Biosystems^®^, TaqMan^®^ Reverse Transcription Reagents kit (Fisher Scientific, Loughborough, UK) was used according to the manufacturer’s instructions to generate cDNA. The Human geNorm Reference Gene Kit with Perfect Probe (Primer Design, Eastleigh, UK) revealed that *RPL 13A* was the most appropriate housekeeping gene for this study. Real-time PCR was performed using SensiFAST™ SYBR green No-rox kit (Bioline, London, UK) with human specific primers for *IL-6*, *IL-8* and *RPL 13A* (Primerdesign, UK; Table A1). Gene expression levels were analysed by two-step quantitative real-time PCR. Data were analysed by the ^ΔΔ^Ct method and normalised to the housekeeping transcript, *RPL 13A* (Primerdesign validated primer).

For protein analysis, cell-free supernatant was collected, and IL-6 and IL-8 levels were analysed using the human DuoSet^®^ ELISA development system (R&D Systems, Abington, UK) according to the manufacturer’s instructions.

### 2.6. Statistical Analysis

Data are presented as mean +/− SEM from data collated across experimental repeats (*n* = 6 per data point, a biological triplicate and repeated experiment). Data were considered significant if *p* < 0.05. One-way ANOVA or two-way ANOVA with Bonferroni post hoc tests and linear regression were used to compare differences as stated. Statistical analysis was performed using GraphPad Prism 5 for Mac OS X (GraphPad Software Inc., San Diego, CA, USA).

## 3. Results

### 3.1. Germination of A. fumigatus Conidia Induces Cytokine Production by Airway Epithelial Cells

**Figure 1:**
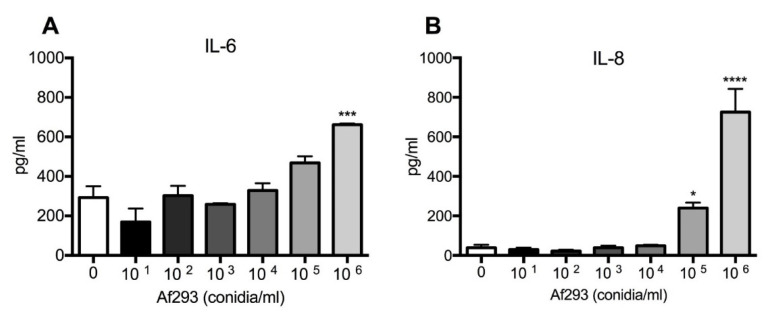
*A. fumigatus* conidia induce IL-6 and IL-8 production in a dose-dependent manner. *A fumigatus* conidia significantly induced (**A**) IL-6 at 10^6^ and (**B**) IL-8 at 10^6^ and 10^5^ conidia/mL compared with the unexposed control. Data represent mean +/− SEM analysed by one-way ANOVA with Bonferroni multiple comparison test. **** *p* < 0.0001; *** *p* < 0.001; * *p* < 0.05 compared with the control.

Bronchial epithelial cell monolayers (16HBE14o-) were exposed to increasing concentrations of *A. fumigatus* conidia (Af293 strain) for 24 h, and levels of IL-6 and IL-8 were assessed by ELISA. Exposure to germinating conidia caused a significant increase in proinflammatory cytokine secretion at 10^5^/mL conidia (IL-8) and 10^6^/mL conidia (IL-6 and IL-8) compared with unexposed control cells (Figure 1A,B). Therefore, for all subsequent experiments, conidia were administered at a concentration of 10^6^/mL.

**Figure 2:**
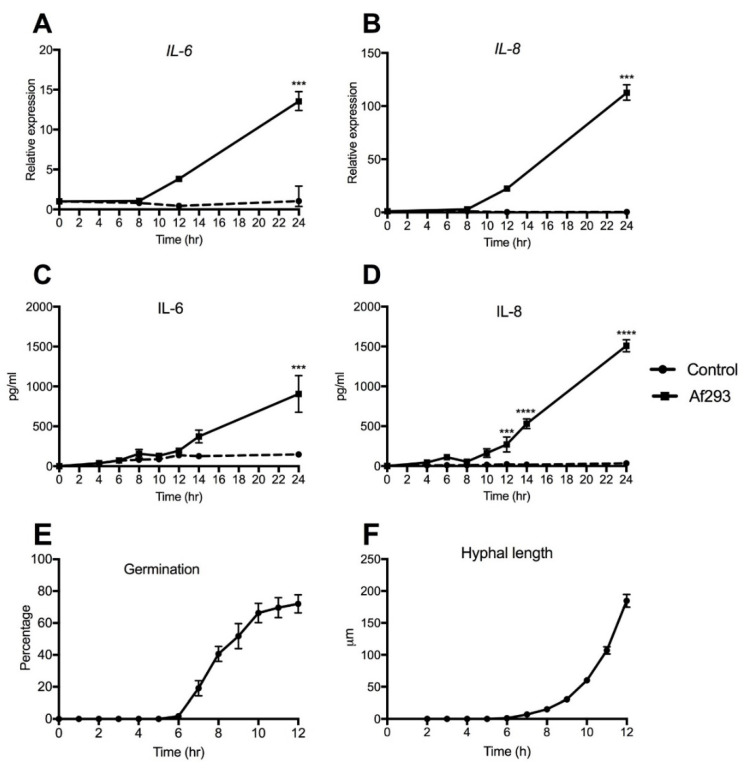
*A fumigatus*-induced cytokine secretion coincides with the onset of conidial germination. *A. fumigatus* significantly upregulated both (**A**) *IL-6* and (**B**) *IL-8* gene expression compared with unexposed control cells. (**C**) IL-6 protein level was significantly increased at 24 h, whereas (**D**) IL-8 reached significance at 12 h and 24 h compared with control. (**E**) Percentage germination and (**F**) hyphal growth of GFP-expressing conidia co-cultured with 16HBE14o- cells showed germination after 6 h, with a rapid increase between 6 and 10 h. Data represent mean +/− SEM; two-way ANOVA with Bonferroni multiple comparison test. **** *p* < 0.0001; *** *p* < 0.001; compared with the control.

To assess the temporal profile of *A. fumigatus*-induced IL-6 and IL-8 production, 16HBE14o- cells were exposed to conidia (strain Af293) and assessed over a 24 h time period. An upregulation of *IL-6* gene expression was found at 8 h, which was 4-fold above the control by 12 h, and 14-fold above the control expression by 24 h (Figure 2A). *IL-8* expression showed a similar upregulation from 8 h, with a 22-fold increase by 12 h and 112-fold increase at 24 h compared with the expression by unexposed control cells (Figure 2B). Protein expression showed a similar trend with the level of both cytokines increasing after 8 h. IL-6 levels were significantly increased by 24 h, whilst IL-8 levels were significantly higher than control at 12 h, 14 h and 24 h post-exposure (Figure 2C,D). In order to relate the temporal secretion of cytokines to the stage-specific growth of *A. fumigatus*, percentage germination and hyphal extension of *A. fumigatus* co-cultured with 16HBE14o- cells was assessed. To aid visualisation, a GFP-expressing strain of *A. fumigatus* was used that demonstrated similar growth kinetics to the Af293 strain (data not shown). Germination commenced from 6 h, with conidial swelling and germlings observed between 6 and 10 h and hyphal growth occurring by 12 h, by which point around 80% of the conidia was germinated (Figure 2E,F). It was not possible to assess germination after this time because of excessive fungal growth and the establishment of a mycelial network. Cytokine gene expression and increased protein production occurred once germination and hyphal extension were established (Figure 2A–D)

### 3.2. Primary Human Airway Epithelial Cells Show Increased Cytokine Production in Response to A. fumigatus

**Figure 3:**
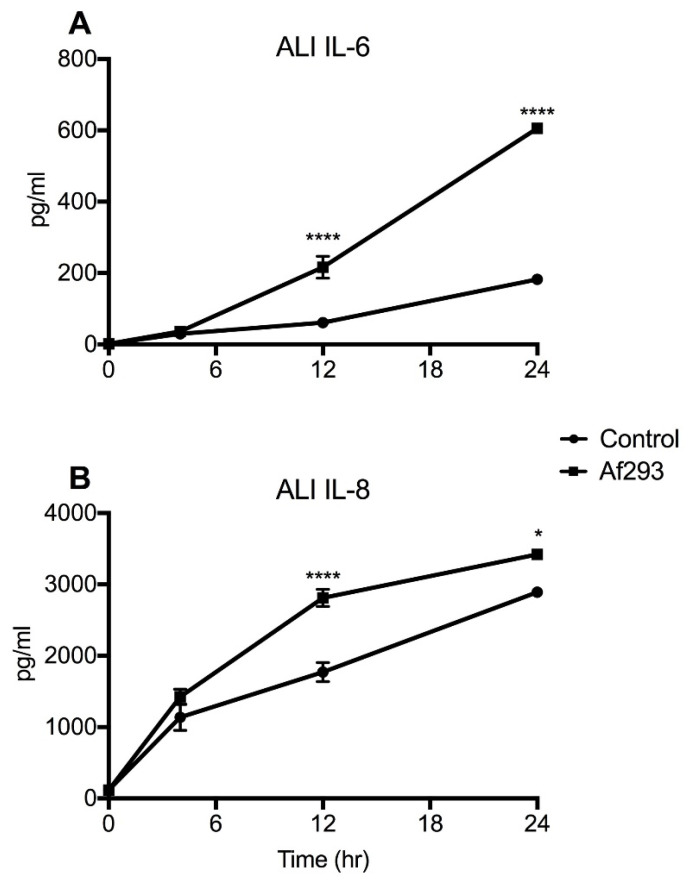
Primary human nasal epithelial cells grown at air–liquid interface show progressive increase in cytokine levels following exposure to *A. fumigatus*. Exposure to *A. fumigatus* conidia caused a significant upregulation of (**A**) IL-6 and (**B**) IL-8 protein levels at 12 h and 24 h, relative to controls. Data represent mean +/− SEM; one-way ANOVA with Bonferroni multiple comparison test. **** *p* < 0.0001, * *p* < 0.05 compared with control.

To determine whether the dynamics of cytokine production in primary human airway epithelial cells were similar to transformed airway cells, primary HNECs were grown at ALI for 14 days and then exposed to *A. fumigatus* conidia (Af293). Primary cells displayed a significant induction of IL-6 at 12 and 24 h post-exposure (Figure 3A). Similarly, in response to Af293, IL-8 levels showed a significant increase at 12 and 24 h relative to the unexposed controls (Figure 3B). These findings suggest that similar trends in cytokine induction in response to conidia were observed between submerged 16HBE14o- and primary airway epithelial cells at ALI.

### 3.3. Proteases Play a Role in A. fumigatus (Af293)-Elicited IL-8 Induction

**Figure 4:**
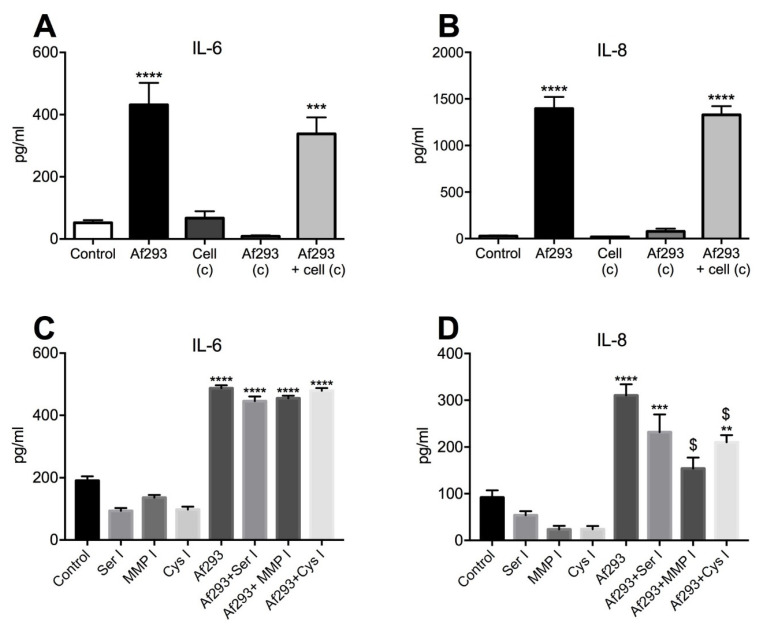
Fungal proteases play a role in Af293 induced IL-8 production. Conditioned media from germinating conidia cultured alone (Af293 c) or from conidia-exposed cells (Af293+cells c) did not induce production of either (**A**) IL-6 or (**B**) IL-8. Protease inhibition had no effect on (**C**) Af293-induced IL-6 production; however, (**D**) MMP inhibition (MMP I) and cysteine protease inhibition (Cys I) significantly reduced IL-8 levels. Data represent mean +/− SEM; one-way ANOVA with Bonferroni multiple comparison test. **** *p* < 0.0001; *** *p* < 0.001, ** *p* < 0.01 compared with unexposed control and $ indicates *p <* 0.0001 for MMP I and *p <* 0.02 for Cys I compared with Af293-exposed cells.

In order to assess the contribution of fungal-derived secreted factors in cytokine induction, 16HBE14o- cells were exposed to conidia for 24 h and conditioned media were collected, filtered and transferred to naïve cells for a further 24 h. Relative to the cytokine levels observed in direct response to conidia, conditioned media derived from conidia-exposed epithelial cells did not induce a further increase of IL-6 or IL-8 in naïve cells over that already present in the conditioned media (Figure 4A,B). Similarly, conditioned media from conidia cultured without cells for 24 h did not induce cytokine production in naïve cells. Protease activity in the conditioned media derived from these cultures was below the level of detection using a universal protease assay (data not shown). To further clarify the contribution of *A. fumigatus* proteases, specific protease inhibitors were added to the cultures. Serine, MMP or cysteine protease inhibitors did not affect the level of IL-6 induced by Af293 conidia (Figure 4C). However, the presence of a MMP inhibitor caused an approximate 2-fold decrease and the cysteine protease inhibitor caused an approximate 1.5-fold decrease in the IL-8 level compared with the no inhibitor control cultures (Figure 4D). Taken together, these findings suggest a potential role for *A. fumigatus* metalloprotease and cysteine proteases in IL-8 induction in response to *A. fumigatus* conidia (Af293).

### 3.4. Secreted A. fumigatus Metalloprotease (A1160+) and Cysteine Proteases Induce IL-8 Production

**Figure 5:**
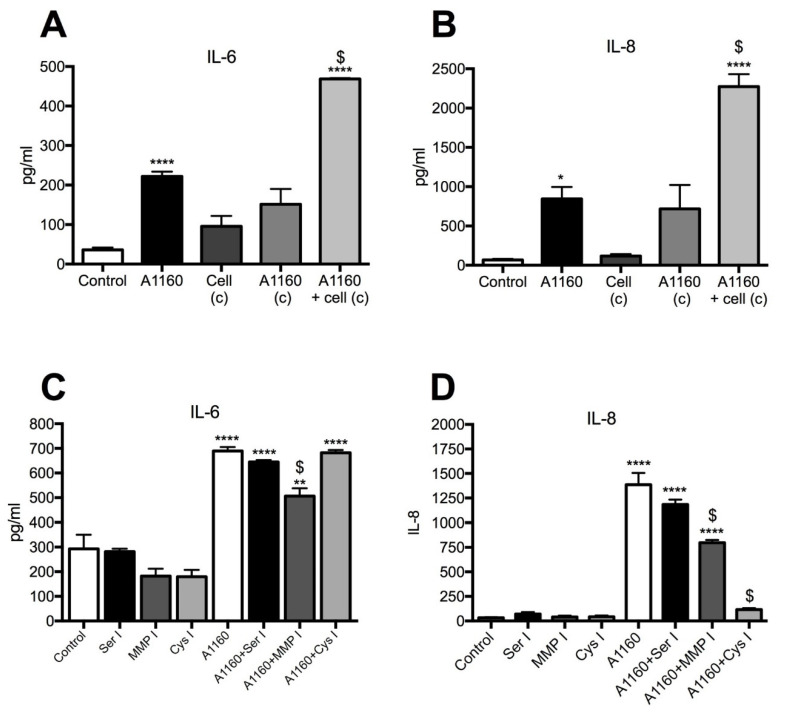
*A. fumigatus* (A1160pyrG+) metalloprotease and cysteine proteases drive cytokine induction. Conditioned media from conidia alone (A1160 c) significantly induced production of both (**A**) IL-6 and (**B**) IL-8, whereas conditioned media from A1160pyrG+-exposed cells (Af1160+cell c) induced a further production of both IL-6 and IL-8. (**C**) Serine and cysteine protease inhibitors showed no effect on IL-6 induction, whilst the MMP inhibitor partially but significantly reduced IL-6 induction. (**D**) Both MMP (MMP I) and cysteine protease (Cys I) inhibitors significantly reduced A1160+-induced IL-8 production. Data represent mean +/− SEM, one-way ANOVA with Bonferroni multiple comparison test. **** *p* < 0.0001; ** *p* < 0.01, * *p* < 0.05 compared with control. $ indicates *p* < 0.01 for MMP I IL-6 reduction compared to A1160+-exposed cells. $ indicates *p* < 0.0001 for IL-8 reduction compared to A1160+-exposed cells.

To determine whether a higher protease-producing strain of *A. fumigatus* induced a greater proinflammatory cytokine response, conidia from *A. fumigatus* strain, A1160pyrG+, were used. As with the Af293 strain, A1160pyrG+ conidia significantly induced IL-6 and IL-8 production by 16HBE14o- cells (Figure 5A,B). Interestingly, conditioned media from cells grown in the presence of conidia (A1160pyrG+) for 24 h significantly induced both IL-6 and IL-8 production in naïve cells, above that induced by directly exposed cells. Furthermore, conditioned media from A1160pyrG+ conidia grown without cells for 24 h significantly induced the production of IL-6 and IL-8 in naïve cells to a similar extent as that from directly exposed cells (Figure 5A,B). However, the protease activity was once again below the level of detection in the conditioned media derived from these cultures in the protease assay used (data not shown). The presence of a serine protease inhibitor again did not reduce production of either cytokine; however, a MMP inhibitor significantly inhibited *A. fumigatus* (A1160pyrG+)-induced IL-6 (approximately 1.3-fold) and IL-8 (approximately 1.7-fold) production. Cysteine protease inhibition did not affect *A. fumigatus* (A1160pyrG+)-induced IL-6 production but dramatically reduced IL-8 levels (11-fold) compared with that produced by conidia-exposed cells. Taken together these findings confirm that *A. fumigatus* metalloproteases and cysteine proteases contribute to IL-8 induction by airway epithelial cells.

**Figure 6:**
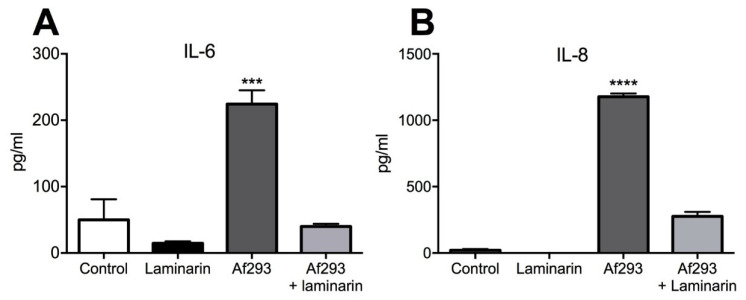
Dectin-1 receptor antagonism inhibits the induction of proinflammatory cytokines. Addition of a Dectin-1 receptor inhibitor, laminarin, to conidia (Af293)-exposed cells for 24 h significantly reduced (**A**) IL-6 and (**B**) IL-8 levels compared with the no laminarin conidia-exposed control. Data represent mean +/− SEM; one-way ANOVA with Bonferroni multiple comparison test. **** *p* < 0.0001, *** *p* < 0.001 compared with the control.

To investigate whether Dectin-1 was also involved in *A. fumigatus* recognition and subsequent cytokine production, the Dectin-1 receptor antagonist, laminarin, was introduced. Blocking Dectin-1 caused a significant (5.6-fold) reduction in IL-6 and a 4.2-fold reduction in IL-8 production compared with conidia-exposed group (Figure 6A,B). These observations suggest a dual role for proteases and Dectin-1-β-glucan moieties in the induction of proinflammatory cytokines by airway epithelium in response to *A. fumigatus*.

## 4. Discussion

In the current study, we found differential induction of IL-6 and IL-8 by airway epithelial cells following exposure to two different strains of *A. fumigatus.* Cytokine induction required at least 8 h of exposure to *A. fumigatus* conidia coinciding with the process of germination and hyphal extension. The extent of epithelial cell response was reduced by the addition of protease inhibitors, indicating that certain proteases play a key role in mediating proinflammatory cytokine response. In addition, conditioned media generated by A1160pyrG+ conidia grown alone was found to induce both IL-6 and IL-8 cytokine production, indicating that secreted proteases were involved. IL-8 was found to be induced by *A. fumigatus* metalloprotease activity, but to a greater extent by cysteine protease activity, whereas IL-6 production was only induced by *A. fumigatus* metalloprotease activity. Thus, the impact of secreted proteases on the proinflammatory response appears highly dependent on the protease secretion profile of the isolate of *A. fumigatus* used. Furthermore, induction of IL-6 and IL-8 production was also mediated by β-glucan recognition by airway epithelial cells as co-incubation with the Dectin-1 receptor antagonist, laminarin, blocked cytokine induction.

Our findings support those of others showing that *A. fumigatus* conidia germination and fungal growth are necessary for proinflammatory cytokine induction [13,14,17,18]. Indeed, Bellanger et al. found that germination and growth, and not conidial internalisation, were responsible for induction of inflammatory cytokines, IL-8, GMCSF and TNF-α, by an alveolar epithelial A549 cell line [19]. Previous studies have suggested that soluble proteolytic factors secreted by growing *A. fumigatus* can induce epithelial cytokine production in vitro [34,36]; although, culture filtrate was used rather than live conidia. Kauffman and colleagues grew a clinical isolate of *A. fumigatus* in a collagenous substrate to generate culture filtrate with high protease activity that induced IL-6 and IL-8 production by A549 cells; although, *A. fumigatus* serine protease activity was proposed to be the main inducing agent, but not metalloprotease or cysteine proteases [37]. The reason for this difference from the current study may be due to the culture filtrate used. In the previous study, it was from *A. fumigatus* cultures grown for 2–5 days, so mature mycelium would have formed, and possibly, an altered protease profile was produced compared with that secreted during germination and early hyphal growth over the first 24–48 h, as in the current study. Moreover, we have previously shown that the profile of proteases released during *A. fumigatus* germination, and early growth depends on the culture substrate present [41]. Thus, isolate Af293 secreted proteases in the presence of complex protein substrates, such as homogenised lung tissue or mucins, but not when grown in Vogel’s minimal media over a 48 h time period [43]. By contrast, the strain A1160pyrG+ exhibited protease activity in Vogel’s minimal media even in the absence of a complex protein [41,42]. *A. fumigatus* metalloprotease activity found in the current study may, in part, be due to Asp f 5 or Mep, which can degrade collagen and elastin [46,47]. The identity of the secreted cysteine protease activity is less clear but may be similar to PalB, a calpain-like, calcium-activated cysteine protease found in *Aspergillus nidulans* [48]. It is worth noting that there are at least 231 putative secreted proteases for the *A. fumigatus* genome, many of which have not yet been identified [49]. Of relevance, Neustadt et al. used free flow electrophoresis and mass spectrometry and found cysteine protease activity in culture filtrate from *A. fumigatus* [50], suggesting that a cysteine protease may be secreted by certain isolates but not others and has yet to be fully characterised. A possible limitation of the data presented is the non-targeted action of protease inhibitors. For instance, epithelial cell-derived MMP and cysteine proteases could contribute to cytokine induction in an autocrine manner and may have also been blocked by protease inhibitors. Furthermore, whilst no morphological impact of protease inhibitors on monolayer integrity was observed, quantification of cell viability and inhibitor dose response may have strengthened the findings. Future studies to characterise the nature of proteases produced by germinating conidia grown in close contact with epithelial cells will require a combination of techniques, such as mass spectrometry, substrate degradation assays and protease inhibitor analysis, to enable the discrimination between human and fungal proteases and whether they are active or inactive.

Our findings suggest that reduced cytokine induction in the presence of protease inhibitors was less pronounced compared with inhibition of Dectin-1-cell wall β-glucan interaction by laminarin, perhaps suggesting that secreted proteases and direct interaction with PPRs by *A. fumigatus* may both play a role, but the importance of each is determined by the culture environment and whether a high or low protease-producing isolate is being assessed. These findings support those of Sun et al. showing that Dectin-1 expression is inducible in bronchial epithelial cells exposed with *A. fumigatus,* and the silencing of Dectin-1 with siRNA resulted in a significant reduction in *IL-8* gene expression [14]. In addition, levels of inflammatory cytokines in bronchioalveolar lavage fluid following *A. fumigatus* airway exposure in mice have been shown to be, in part, Dectin-1 dependent [30,31], with Dectin-1 knockout mice displaying increased susceptibility to aspergillosis, suggesting an essential role for this PPR in host defence [32]. Therefore, it is likely that host–pathogen relationships are multiple and complex, and taken together, the induction of proinflammatory cytokines and resultant airway inflammation occur when fungal cell wall components are exposed, and proteases are secreted in an isolate-dependent manner. Furthermore, it may be that the mechanism of cytokine induction is dynamic, with early induction dependent on germinating conidia cell wall component exposure, whilst later, secretion of proteases from invading hyphae become prominent. Elucidating the mechanisms by which different *A. fumigatus* isolates drive inflammatory responses will provide a better understanding of differential disease aetiology and contribute to the development of novel treatment strategies.

## Data Availability

Data are contained within the article.

## Appendix A

**Table A1:** Forward and reverse primer sequences for *IL-6* and *IL-8*.

Gene	Sense	Antisense
*IL-6*	GCAGAAAACAACCTCAACCTT	ACCTCAAACTCCAAAAGACCA
*IL-8*	CAGAGGGTTGTGGAGAAGTTT	ATGAAGTGTTGAAGTAGATTTGCT
